# Procalcitonin to allow early detection of sepsis and multiple organ failure in severe multiple trauma: beware of some confounders

**DOI:** 10.1186/s13054-020-2729-6

**Published:** 2020-01-07

**Authors:** Patrick M. Honore, Christina David, Rachid Attou, Sebastien Redant, Andrea Gallerani, David De Bels

**Affiliations:** ICU Department, Centre Hospitalier Universitaire Brugmann, Place Van Gehuchtenplein 4, 1020 Brussels, Belgium

AlRawahi et al. concluded that sepsis and multiple organ failure (MOF) are the predominant cause of late death in severe multiple trauma (MT) [1]. They suggested that repeated measures of procalcitonin (PCT) during disease course may allow for early recognition of septic complications and detection of multiple organ failure (MOF), resulting in earlier therapeutic decisions and an impact on survival and improve outcomes [1].

We would like to make some comments. A recent meta-analysis evaluating more than 26,000 MT patients revealed a pooled incidence of acute kidney injury (AKI) in MT of more than 20% [2]. In addition, in a further breakdown of the A KI stages, over 40% of these patients were classified with more severe forms of AKI (RIFLE I or F or stages 2–3) [2] suggesting a probable use of renal replacement therapy (RRT) between 5 and 10% [2]. This incidence could be even higher (up to 20%) if MT is complicated by sepsis and MOF [3]. If we apply the same trends for the study of AlRawahi et al., the incidence of RRT in MT with sepsis and MOF could have an impact upon the reliability of the PCT level under those conditions. PCT has an approximate molecular weight of 14.5 kDa [4]. The contemporary continuous RRT (CRRT) membranes are able to remove molecules as large as 35 kDa [4]. Hence, most of the PCT mass will be eliminated by convective flow [4], but adsorption also contributes to the elimination if using new highly adsorptive membranes (HAM) [5]. Accordingly, an imbalance between the use of CRRT in the two cohorts (MT alone or MT with sepsis and MOF) will have an important impact upon the values of PCT in each cohort but more so in the MT cohort with sepsis and MOF. PCT levels may therefore be affected not only by the complications of MT but also by the incidence of RRT. In conclusion, we believe there is a critical need for a future study with a focus on the performance of the currently known biomarkers among patients receiving CRRT [5].

## Data Availability

Not applicable.

## Abbreviations

AKI: Acute kidney injury
CRRT: Continuous renal replacement therapy
HAM: Highly adsorptive membranes
MOF: Multiple organ failure
MT: Multiple trauma
PCT: Procalcitonin
RRT: Renal replacement therapy
